# Primary benign intratracheal schwannoma: A case report and review of the literature

**DOI:** 10.1002/rcr2.1241

**Published:** 2023-10-31

**Authors:** Shahab Rafieian, Reza Ershadi, Hesam Amini, Alireza Samimiat, Mohsen Gholinataj Jelodar, Matin Vahedi

**Affiliations:** ^1^ Department of Thoracic Surgery, Imam Khomeini Hospital Complex Tehran University of Medical Sciences Tehran Iran; ^2^ Department of Surgery, Sina Hospital Tehran University of Medical Sciences Tehran Iran; ^3^ Department of Internal Medicine Shahid Sadoughi University of Medical Sciences Yazd Iran

**Keywords:** resection, schwannoma, trachea

## Abstract

Shwannomas are rare benign tumours especially in tracheal. A 16‐year‐old male presented with a chronic cough, and a thoracic CT scan revealed a pedunculated tumour measuring approximately 11 × 13 mm in size, located 22 mm away from the main carina. Tissue sample was obtained via rigid bronchoscopy and cryobiopsy, and the pathological analysis confirmed the diagnosis of a benign nerve sheath tumour consistent with schwannoma. The patient subsequently underwent resection of the tumour and tracheal anastomosis. Schwannomas are uncommon pulmonary tumours that typically occur in adults, with a higher incidence among females. The presenting symptoms vary depending on the size and location of the tumour. Treatment options include therapeutic bronchoscopy or surgical resection, with the choice of approach based on tumour characteristics (pedunculated or sessile), preoperative surgical risk, and risk of recurrence. The prognosis is generally favourable, with a low risk of recurrence and excellent outcomes.

## Graphical abstract

Schwannomas are benign tumours that originate from Schwann cells. Primary trachea tumours are uncommon. Here we report a case of primary schwannoma of the trachea in a 16 year‐old male patient.

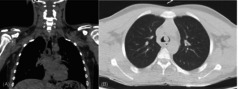

## INTRODUCTION

Schwannomas are benign tumours that originate from Schwann cells.^1^ These tumours can grow in the peripheral nerves, mediastinum, posterior mediastinum, posterior spinal nerve roots, and Cerebellopontine angle (CPA).^2^

Primary trachea tumours are uncommon. Among these tumours, 75% are composed of squamous cell carcinoma and adenoid cystic carcinoma. The remaining tumours have diverse histological subtypes and can be benign, malignant, or of intermediate grade.^3^

Neurogenic tumours of the tracheobronchial tumours, such as schwannomas and neurofibromas, are exceedingly rare.^4^

The purpose of this report is to present a case of primary schwannoma of the trachea.

## CASE PRESENTATION

**FIGURE 1:**
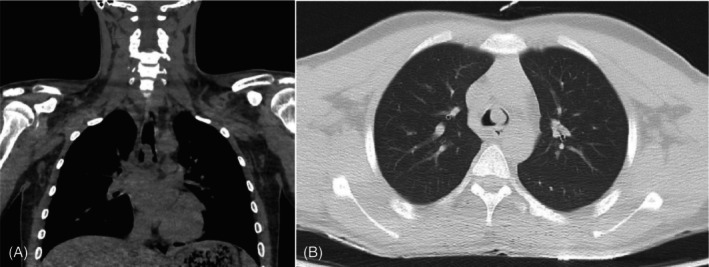
Chest computed tomography scan. Coronal (A) and axial (B) image a pedunculated polypoid mass measuring approximately 11 × 13 mm in cross‐sectional diameter, situated in the distal trachea and extending over a cranio‐caudal length of 21 mm.

**FIGURE 2:**
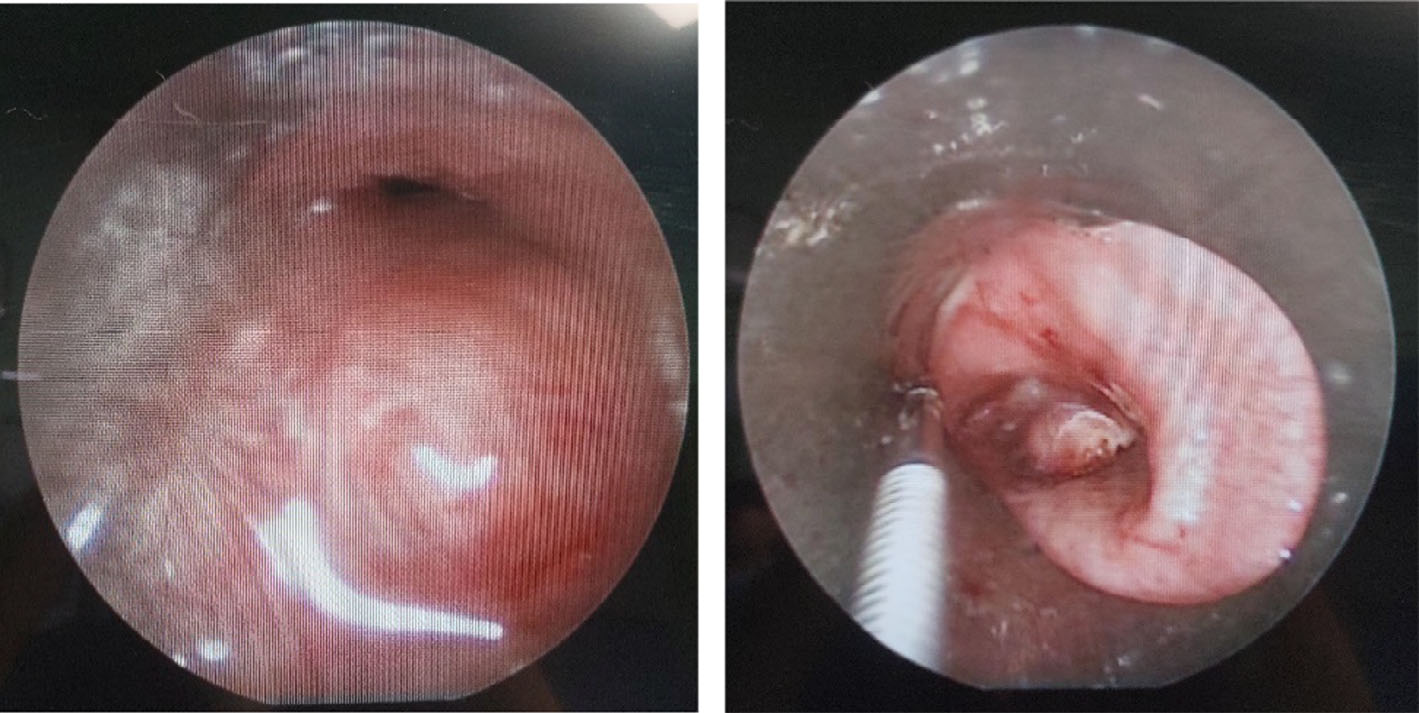
Bronchoscopy revealed a pedunculated polypoid lesion bove the carina and deviating into the right main bronchus with smooth and intact surface.

**FIGURE 3:**
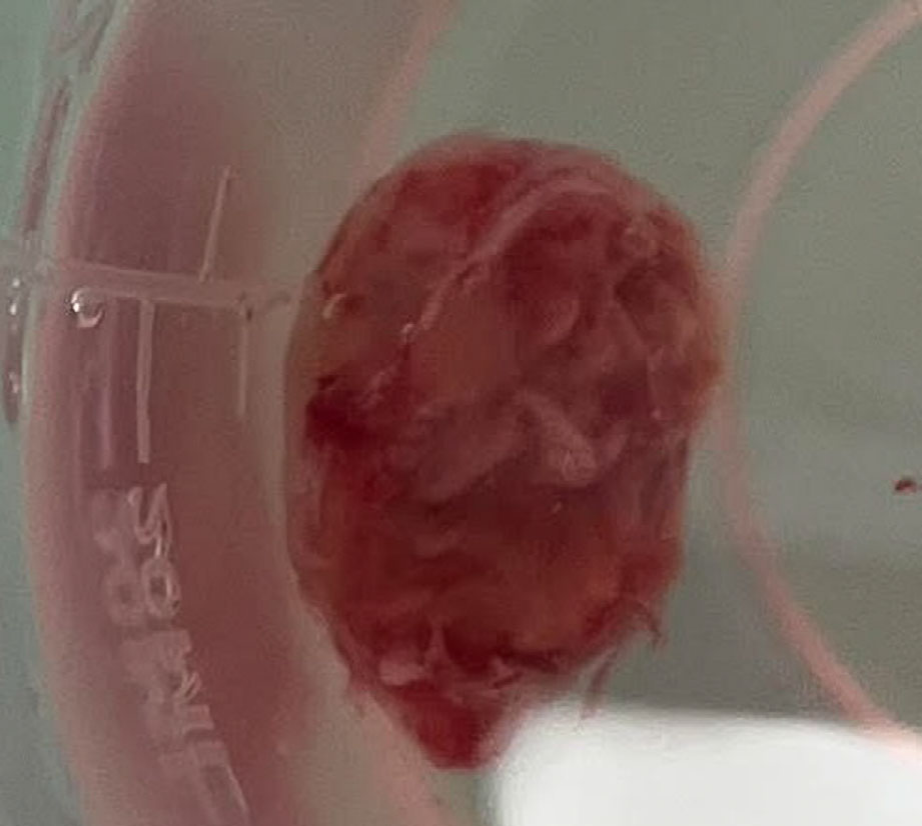
Gross view from cryobiopsy of tracheal tumour.

A 16‐year‐old male with an unremarkable medical history presented to the thoracic surgery clinic with a chief complaint of a non‐productive cough that had been persistent for approximately 3 months despite the use of cough suppressant medications. The patient was asymptomatic for weight loss, fever, diaphoresis, anorexia, or hemoptysis. There was no history of tobacco or substance use, drug or food allergies, familial respiratory or malignant diseases, or any recent close contact with an ill individual. The patient demonstrated stable vital signs and a normal cardiac and pulmonary examination. Sputum cultures were negative for mycobacteria on three separate occasions. Chest CT scan revealed a pedunculated polypoid mass measuring approximately 11 × 13 mm in cross‐sectional diameter, situated in the distal trachea and extending over a cranio‐caudal length of 21 mm (Figure 1). The lower edge of the mass terminated just above the carina, arising from the right tracheal wall at the 9 o'clock position, approximately 22 mm above the carina. The upper edge of the mass was situated approximately 8.5–9 cm from the vocal cords, and no pathological lesions were identified in other regions of the lung, mediastinum, or chest wall. Based on the findings, the patient was admitted to the thoracic surgery department and underwent rigid bronchoscopy under general anaesthesia by a pulmonologist. The bronchoscopy revealed a pedunculated polypoid lesion arising from a location above the carina and deviating into the right main bronchus. The surface of the lesion appeared smooth and intact (Figure 2). A tissue sample was obtained from the lesion using cryobiopsy (Figure 3). No endobronchial lesions were identified in other areas.

**FIGURE 4:**
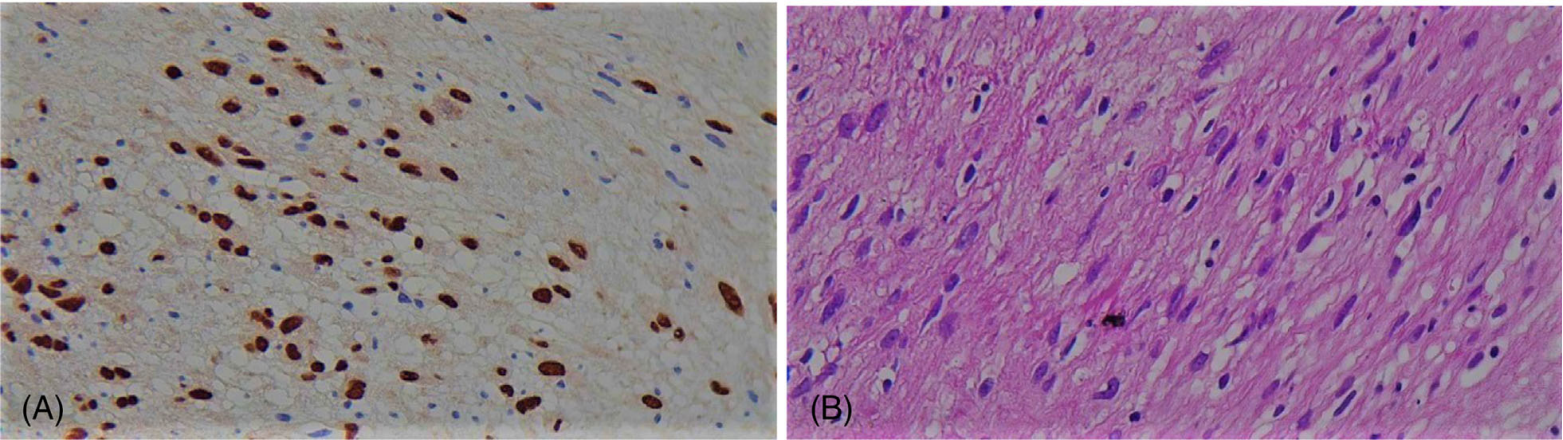
Immunohistochemical staining with SOX10 marker, the cell nuclei are strongly stained (A). Microscopic image of the lesion with 40 magnification showing spindle‐shaped and wavy cells without pleomorphism, mitosis or necrosis (B) consistent with a diagnosis of schwannoma.

The pathological examination of the lesion revealed a benign nerve sheath tumour that stained positively for SOX10 on immunohistochemistry, consistent with a diagnosis of schwannoma (Figure 4).

According to the pathology report, it was decided to remove the tumour. Considering the young age of the patient and more surgical comorbidities, the initial plan for the patient was to remove the mass by endoscopic method, but considering the bronchoscopy and the wide base of the mass, the surgical plan was preferred for the patient.

**FIGURE 5:**
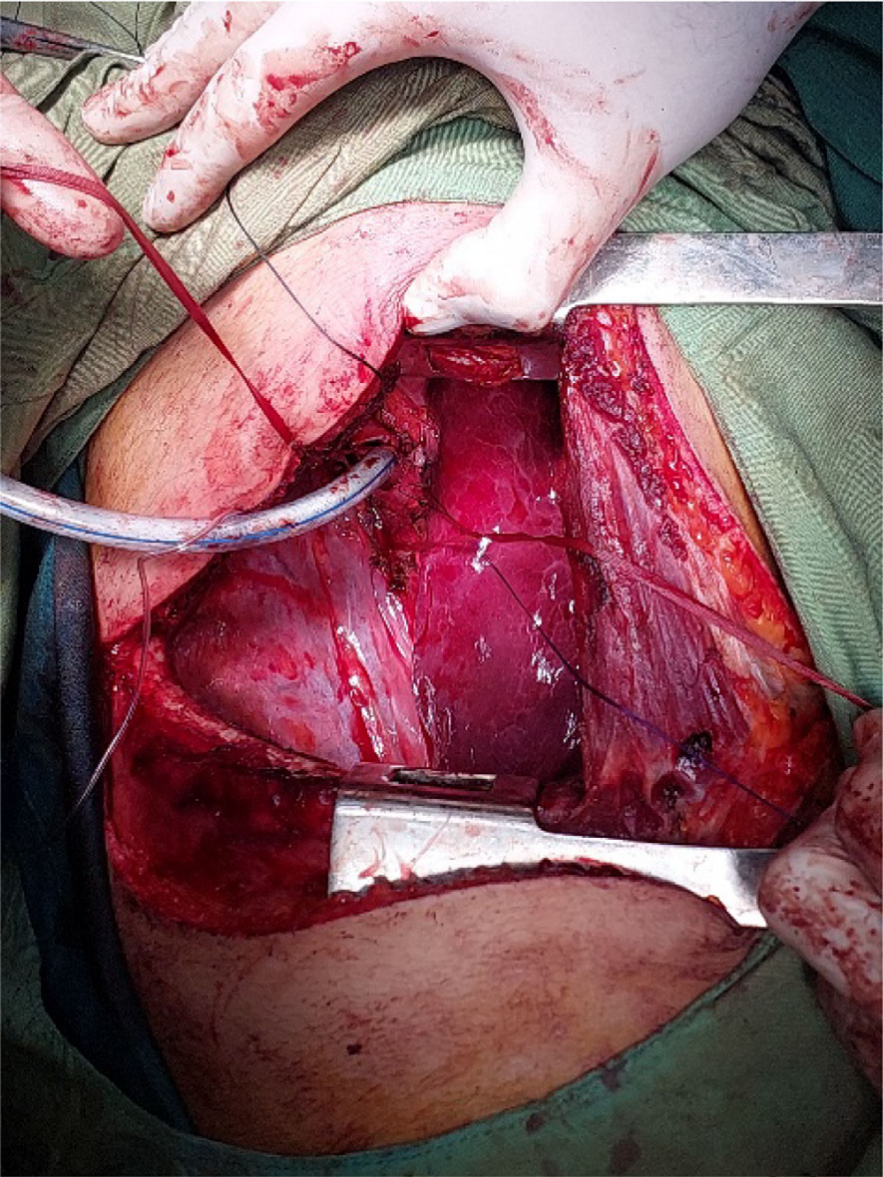
Tracheal resection by thoracotomy.

First, the patient was intubated by an anesthesiologist with a simple endotracheal tube. The patient was placed in the left lateral decubitus position. Following the principles of sterilization, a right posterolateral thoracotomy incision was made in the fifth intercostal space and the trachea was explored. The carina was exposed, then a sterile endotracheal tube was inserted into the left main bronchus and the patient was ventilated. Then, the trachea was cut transversely above the tumour site, and resection of the trachea along with the tumour was performed approximately 3 cm long (Figure 5). Anastomosis of the involved part was performed using 3.0 PDS thread. Then the patient was transferred to the intensive care unit as an extubation.

One week later, the patient underwent fiberoptic bronchoscopy, and the anastomosis site was completely normal. The patient was discharged from the hospital on the eighth day after the operation.

**FIGURE 6:**
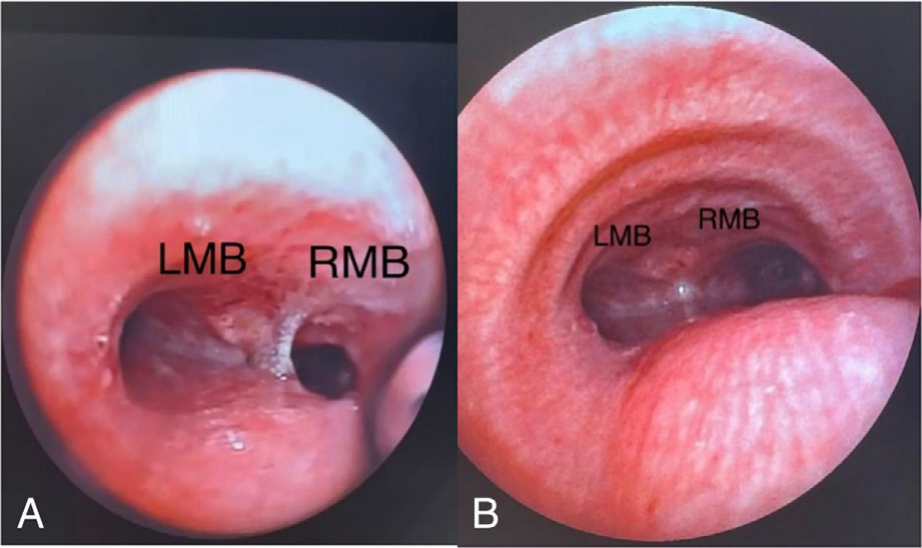
Fiberoptics bronchoscopy 1 month(A) and 3 months (B) after discharging.

Also, for follow up, the patient underwent bronchoscopy 1 month and 3 months after discharge (Figure 6).

## DISCUSSION

Tracheal schwannoma is an infrequent presentation, with the majority of reported cases found in the lungs and bronchi.^2^

Schwannomas usually present as solitary lesions with a complete capsule, are connected to the nerve sheath, and may demonstrate vascularity with multiple small and separate vessels.^2^, ^5^, ^6^, ^7^

Schwannoma is rarely associated with von Recklinghausen's disease (Neurofibromatosis type 1), and malignant transformation of the tumour is extremely infrequent.^5^, ^6^, ^7^, ^8^

Kashara et al^8^ have proposed a classification system for pulmonary schwannomas. They classified pulmonary schwannomas into two groups based on their location: central lesions located in the hilum and anterior regions of the bronchi that are visible on bronchoscopy, and peripheral lesions that are not visible on bronchoscopy and can be observed as nodules on chest x‐ray or CT scan. The central type is further divided into two subtypes: Type 1, which is confined to the intraluminal space, and type 2, which can exist in both interaluminal and extraluminal spaces.

In the literature review, four cases of this rare disease were reported and are referred to in Table 1.

**TABLE 1:** Four cases of this rare disease were reported in previous literature.

	Horovitz et al.^6^	Righini et al.^9^	Tang et al.^10^	Xiahui et al.^2^
Year	1950–1983	1950–2003	1950–2005	1950–2013
Number of cases	13	23	34	51
Adults	11 (84.6%)	19 (82.6%)	27 (80%)	40 (78.4%)
Female:Male	(1 Case unknown) 7:5	(5 Case unknown) 14:4	(1 Case unknown) 18:13	(1 Case unknown) 30:20
Location in the tracheal	Inferior > Superior > Middle	NA	Middle > Superior > Inferior	Middle > Superior > Inferior
Race	Caucasian > Others	NA	NA	Asia > North America > Europe
Common symptoms	Cough, wheezing, dyspnea, and hemoptysis	Upper airway obstruction with predominant dyspnea	Symptoms of airway obstruction such as cough, wheezing, and dyspnea	Cough, wheezing, and dyspnea
Uncommon symptoms	Fever and chest pain	Hemoptysis	Hoarseness, hemoptysis, sudden dyspnea	Hemoptysis, hoarseness, and chest pain
Signs	NA	NA	Pneumomediastinum, subcutaneous emphysema, and fever	Wheezing (29% stridor)
Assessment	Fluoroscopy, x‐ray, tomography, chest CT scan, bronchoscopy	Chest CT scan, MRI (1case), bronchoscopy	Pulmonary function test, chest CT scan, MRI, bronchoscopy	Pulmonary function test, chest CT scan, bronchoscopy
Delay to diagnosis (m)	10–15	10–15	NA	17
Tumour size	NA	NA	NA	1–4 cm
Endoscopic removal	5 (38%)	NA	NA	19 (37%)
Surgical removal	8 (62%)	NA	NA	33 (71%)
Recurrence after endoscopic removal	1 (20%)	1 (4%)	3 (8.6%)	4 (21%)
Recurrence after endoscopic surgical	0 (0%)	0 (0%)	0 (0%)	0 (0%)

The clinical manifestations of intratracheal schwannoma depend on the size, location, and degree of obstruction caused by the tumour.^2^ Due to the rarity of this tumour and its nonspecific clinical symptoms, there is an average delay of approximately 17 months from the onset of symptoms to diagnosis.^2^, ^7^

Schwannoma of the intratracheal is a disease of adults and is more common in women.^2^, ^7^, ^9^

The location of tracheal schwannomas is frequently in the distal third of the tracheal, followed by the proximal third and middle third, respectively.^2^, ^5^, ^9^

Diagnosis is confirmed by bronchoscope sampling, and CT scan helps determine tumour size and extent. Other evaluations include spirometry, which demonstrates fixed obstruction of the upper airway on volume‐flow curve.^9^, ^10^

PET/CT imaging reveals increased uptake in the tumour, although routine use is not recommended.^11^

In our patient, the purpose of performing CT scan was to evaluate the presence of nodules in the lung parenchyma, in addition to assessing the tracheal and the extent of the tumour.

Schwannoma of the tracheal can be treated by several methods, including primary tumour excision or endoscopic treatment using laser, snare resection, argon plasma coagulation (APC), CO2 laser, electrocautery, cryotherapy, with or without endoscope and microdebrider. The choice of surgical approach should be determined by the clinical manifestations of the tumour (pedunculated versus flat), the risk of tracheal resection, and the presence or absence of extratracheal extension.^12^

In our patient, due to the low risk of surgery and incomplete tumour removal by cryobiopsy, the decision was made to perform tracheal resection.

In patients with pedunculated lesions without extratracheal extension or in individuals with high surgical risk, the tumour can be removed using endoscopic techniques. However, tumour recurrence occurs in a quarter of patients.^2^ The time to recurrence varies and even in one case, recurrence occurred with a delay of 12 years.^6^

Due to the slow growth rate of these tumours, bronchoscopic follow‐up every year is recommended. In another group of patients with flat tumours, where the risk of surgery is low or there is extratracheal extension, surgical removal is preferred. Recurrence has not been reported in this group.^2^

The prognosis for patients with schwannoma is excellent, whether treated surgically or endoscopically.^2^

## AUTHOR CONTRIBUTIONS

Hesam Amini and Alireza Samimiat gathered and interpreted the patient data regarding history and physical examination. Shahab Rafieian and Reza Ershadi wrote the manuscript. Matin Vahedi supervised and designed the project. Shahab Rafieian and Matin Vahedi perform surgery on the patient. As, Hesam Amini and Mohsen Gholinataj Jelodar contributed to the discussion of the research. All the authors read, revised and approved the final manuscript.

## CONFLICT OF INTEREST STATEMENT

None declared.

## ETHICS STATEMENT

The authors declare that appropriate written informed consent was obtained for the publication of this manuscript and accompanying images.

## Data Availability

The data that support the findings of this study are available on request from the corresponding author. The data are not publicly available due to privacy or ethical restrictions.

## References

[1] Lina G , Pengguo H , Zhihua X , Jianxin W , Baoqin B , Mingyue Z , et al. Tracheobronchial schwannoma: a case report and literature review. J Int Med Res. 2023;51(1):3000605221149891. 36708207 10.1177/03000605221149891 PMC9893080

[2] Ge X , Han F , Guan W , Sun J , Guo X . Optimal treatment for primary benign intratracheal schwannoma: a case report and review of the literature. Oncol Lett. 2015;10(4):2273–2276. 26622833 10.3892/ol.2015.3521 PMC4579820

[3] Grillo HC , Mathisen DJ . Primary tracheal tumors: treatment and results. Ann Thorac Surg. 1990;49(1):69–77. 2153371 10.1016/0003-4975(90)90358-d

[4] Xu L‐T , Sun Z‐F , Li Z‐J , Wu L‐H , Zhang Z‐Y , Yu X‐Q . Clinical and pathologic characteristics in patients with tracheobronchial tumor: report of 50 patients. Ann Thorac Surg. 1987;43(3):276–278. 3827370 10.1016/s0003-4975(10)60611-x

[5] Beheshti J , Mark EJ , Grillo H . Mesenchymal tumors of the trachea. Surgery of the trachea and bronchi. Vol 86. London: BC Decker Inc.; 2004. p. 97.

[6] Horovitz AG , Khalil KG , Verani RR , Guthrie AM , Cowan DF . Primary intratracheal neurilemoma. J Thorac Cardiovasc Surg. 1983;85(2):313–317. 6823150

[7] Weiner DJ , Weatherly RA , DiPietro MA , Sanders GM . Tracheal schwannoma presenting as status asthmaticus in a sixteen‐year‐old boy: airway considerations and removal with the CO2 laser. Pediatr Pulmonol. 1998;25(6):393–397. 9671167 10.1002/(sici)1099-0496(199806)25:6<393::aid-ppul7>3.0.co;2-i

[8] Kasahara K , Fukuoka K , Konishi M , Hamada K , Maeda K , Mlkasa K , et al. Two cases of endobronchial neurilemmoma and review of the literature in Japan. Intern Med. 2003;42(12):1215–1218. 14714962 10.2169/internalmedicine.42.1215

[9] Righini CA , Lequeux T , Laverierre M‐H , Reyt E . Primary tracheal schwannoma: one case report and a literature review. Eur Arch Oto‐Rhino‐Laryngol Head Neck. 2005;262:157–160. 10.1007/s00405-004-0778-0 15060831

[10] Dorfman J , Jamison BM , Morin JE . Primary tracheal schwannoma. Ann Thorac Surg. 2000;69(1):280–281. 10654537 10.1016/s0003-4975(99)01195-9

[11] Miyake KK , Nakamoto Y , Kataoka TR , Ueshima C , Higashi T , Terashima T , et al. Clinical, morphologic, and pathologic features associated with increased FDG uptake in schwannoma. Am J Roentgenol. 2016;207(6):1288–1296. 27657364 10.2214/AJR.15.14964

[12] Rusch V , Schmidt R . Tracheal schwannoma: management by endoscopic laser resection. Thorax. 1994;49(1):85–86. 8153948 10.1136/thx.49.1.85 PMC474110

